# Does depression diagnosis and antidepressant prescribing vary by location? Analysis of ethnic density associations using a large primary-care dataset

**DOI:** 10.1017/S0033291715002913

**Published:** 2016-02-16

**Authors:** P. Schofield, J. Das-Munshi, R. Mathur, P. Congdon, S. Hull

**Affiliations:** 1 Division of Health & Social Care Research, Faculty of Life Sciences & Medicine, King's College London, Addison House, Guy's Campus, London, UK; 2 Institute of Psychiatry, Psychology & Neuroscience, King's College London, UK; 3 Centre for Primary Care and Public Health, Queen Mary University of London, UK

**Keywords:** Antidepressants, depression, ethnicity

## Abstract

**Background:**

Studies have linked ethnic differences in depression rates with neighbourhood ethnic density although results have not been conclusive. We looked at this using a novel approach analysing whole population data covering just over one million GP patients in four London boroughs.

**Method:**

Using a dataset of GP records for all patients registered in Lambeth, Hackney, Tower Hamlets and Newham in 2013 we investigated new diagnoses of depression and antidepressant use for: Indian, Pakistani, Bangladeshi, black Caribbean and black African patients. Neighbourhood effects were assessed independently of GP practice using a cross-classified multilevel model.

**Results:**

Black and minority ethnic groups are up to four times less likely to be newly diagnosed with depression or prescribed antidepressants compared to white British patients. We found an inverse relationship between neighbourhood ethnic density and new depression diagnosis for some groups, where an increase of 10% own-ethnic density was associated with a statistically significant (*p* < 0.05) reduced odds of depression for Pakistani [odds ratio (OR) 0.81, 95% confidence interval (CI) 0.70–0.93], Indian (OR 0.88, CI 0.81–0.95), African (OR 0.88, CI 0.78–0.99) and Bangladeshi (OR 0.94, CI 0.90–0.99) patients. Black Caribbean patients, however, showed the opposite effect (OR 1.26, CI 1.09–1.46). The results for antidepressant use were very similar although the corresponding effect for black Caribbeans was no longer statistically significant (*p* = 0.07).

**Conclusion:**

New depression diagnosis and antidepressant use was shown to be less likely in areas of higher own-ethnic density for some, but not all, ethnic groups.

## Introduction

Black and minority ethnic (BME) groups have been consistently shown to have higher rates of severe mental illness compared to the rest of the population (Bourque *et al.* 2011; Veling, 2013). However, this is not as clear for common mental disorders, such as depression, where studies have often shown mixed results (Tarricone *et al.* 2012). Furthermore, UK studies suggest rates of antidepressant use are actually much lower in these groups (Cornwell & Hull, 1998; Cooper *et al.* 2013). A number of studies have related ethnic health differences to area ethnic density, a concept that has attracted considerable research interest in recent years (Shaw *et al.* 2012). An ethnic density effect is proposed whereby members of ethnic minority groups are at less risk of mental ill health if they live in areas with a greater proportion of their own ethnic group. While this is most clearly demonstrated for severe mental illness there is some, albeit limited, evidence that this also applies to anxiety and depression (Halpern & Nazroo, 2000; Propper *et al.* 2005; Pickett *et al.* 2008; Das-Munshi *et al.* 2010) and antidepressant use (Hull *et al.* 2001; Walters *et al.* 2008; Morrison *et al.* 2009; Spence *et al.* 2014). It has been argued, this effect may in part be explained by acculturation bias, where those less likely to adopt the norms of the majority culture tend to cluster together, although there is currently only limited evidence for this (Halpern & Nazroo, 2000; Gonzalez *et al.* 2010).

Studies of neighbourhood effects on common mental disorders have typically been based on relatively small samples and, it is argued, this may explain the lack of consistent findings when compared with studies looking at severe mental illness (Shaw *et al.* 2012). A further problem is that most studies have looked at effects at a relatively broad area level, such as ward or census middle super output area (MSOA), and may therefore fail to detect processes that occur at a more detailed local neighbourhood level, such as the census lower super output area (LSOA) (Mohan *et al.* 2005). In the UK depression is predominantly treated in primary care (NICE, 2004) and, with the increasing availability of large datasets of General Practitioner (GP) records, it should therefore be possible to investigate these questions at this greater level of detail. However, there are recognized limitations to the use of GP records to determine depression diagnosis. The Quality and Outcomes Framework (QOF) requirements for GPs to follow-up certain diagnoses can act as a disincentive to formally code depression and often GPs will enter individual symptoms only to avoid this (Rait *et al*. 2009; Kendrick *et al*. 2015). One solution is to examine, in parallel, an alternative proxy measure of depression such as antidepressant prescribing, that is not subject to the same kind of bias. While a small number of studies using GP records have looked at ethnic density and antidepressant prescribing these have been at practice level only making it difficult to conclude that effects also apply at patient level (Hull *et al.* 2001; Walters *et al.* 2008; Morrison *et al.* 2009; Spence *et al.* 2014).

Using a large database of GP patient records we were able to examine both recent (past year) depression diagnosis and antidepressant use for a range of ethnic groups at a detailed neighbourhood level. The dataset covered four ethnically diverse London boroughs: Lambeth, Hackney, Tower Hamlets and Newham; a total practice population of just over 1 million people. This includes the largest UK concentration of Bangladeshi people, in Tower Hamlets, the second largest UK black Caribbean population, in Lambeth, and also large Indian, Pakistani and black African populations (Office for National Statistics, 2011). This allowed us to assess: first, ethnic differences in antidepressant use and recent depression diagnosis; second, the extent to which this is related to area ethnic density; and third, whether any ethnic density effect is, in turn, related to a measure of acculturation.

## Method

### Data source

GP health records for all patients registered in Lambeth, Hackney, Tower Hamlets and Newham GP practices were extracted on 31 October 2013 for 47 out of 48 practices in Lambeth, 41/43 in Hackney, 64/64 in Newham and 37/37 in Tower Hamlets. One Lambeth practice was unwilling to share records and two Hackney practices used an incompatible electronic records system.

### Outcome

We looked at new diagnosis of depression as recorded in the patient's record at any time in the year prior to the date of extraction. This was based on the standard QOF depression Read codes for 2013/2014. We also looked at any antidepressants prescribed in the same period. We excluded those drugs likely to have a dual indication for prescribing, in addition to a mental health indication. For example, amitriptyline was excluded as it is often used specifically for pain control (see Supplementary Appendix S2 for full list of excluded drugs).

### Predictors

We used patients’ self-declared ethnicity mapped to the following census (2011)-defined ethnic groups: white British, Indian, Pakistani, Bangladeshi, black Caribbean and black African. As information on mixed ethnicity was not consistently available we included only those defined as belonging specifically to the above ethnic groups. Ethnic density, for each corresponding group, was defined as the percentage of people from that group living within each census LSOA as determined using 2011 Census data (Office for National Statistics, 2011). As a proxy for acculturation we used whether English was recorded as the main spoken language. Neighbourhood deprivation, has been shown to be related to both depression outcome and ethnic density (Mair *et al.* 2008) and is therefore a potential confounder. We therefore adjusted for area deprivation using the index of multiple deprivation (IMD 2010; Department for Communities and Local Government, 2011).

### Further inclusion and exclusion criteria

We included all patients in the above ethnic groups along with white British patients (as a comparison group), aged between 16 and 64 years, and registered with a GP practice for at least a year. We excluded patients from unusually small practices (<750 registered patients) as these are likely to be highly atypical (Ashworth *et al.* 2013). We also excluded one practice with unusually low prescribing rates (<0.5%) as this was likely due to coding error. Patients from the City of London financial district were also excluded, as these were typically registered in the area for work purposes rather than local residency.

### Ethical approval

London Bridge research ethics committee agreed that this study does not require ethical approval as it is based on non-identifiable data and results are published in aggregate form only.

### Statistical analysis

We calculated age/gender standardized rates for antidepressant use and new depression diagnosis for each ethnic group. We then examined the effect, on both outcomes, of interactions between ethnic group and own-group ethnic density; adjusting for age, gender and area deprivation. Analysis was carried out using a multilevel model incorporating random effects to account for clustering at neighbourhood and GP practice levels. GP practice effects were incorporated in the model because rates of both antidepressant prescribing and depression diagnosis have been shown to vary considerably by GP practice (Sartorius *et al.* 1996). The relationship between neighbourhood and GP practice is complex as patients from the same neighbourhood may attend a variety of different practices, and each practice covers a large number of neighbourhoods. By using a cross-classified multilevel model we could account for this complex structure and therefore disentangle the effect of living in a particular neighbourhood from the effect of attending a particular GP practice (Dunn *et al.* 2015).

We repeated the ethnic density analysis, including an indicator for whether English was the patient's main language. This included some missing data (17% of BME patients had language missing) and we substituted ‘English main spoken language’ where no information was provided, on the assumption that GPs would be more likely to enter patient's language preference for BME patients if this were not English. We then conducted a sensitivity analysis, where we tested the effect this assumption might have on the results, coding all missing language data as, conversely, ‘English not main language’ and re-analysing the data to see if this made any difference to our conclusions.

The descriptive part of the analysis was carried out using Stata v. 13 (Stata Corporation, 2013) and regression modelling was carried out using the lme4 package in R (R Core Team, 2014; Bates *et al.* 2015).

## Results

We were able to collect data for 4 10 541 patients meeting the main study criteria of whom 2 48 821 were in the above BME groups. Of these 2 05 983 (84%) included information about main spoken language with this missing for 18% of Indians, 21% Pakistanis, 13% Bangladeshis, 17% black Caribbeans and 21% black Africans in the sample.

We found clear ethnic differences in rates of new depression diagnosis (Table 1) after adjusting for age and gender. White British patients were most likely to be recently diagnosed (3.5%) compared with 2.2% black Caribbeans, 1.5% Bangladeshis, 1.2% Pakistanis and 0.9% of Indian patients. Antidepressant use was again highest for white British patients (8.1%), then Bangladeshis (4.4%), black Caribbeans (3.7%), Pakistanis (3.2%) and Indian and African patients equally had the lowest rate (2.1%).

**Table 1.** Description of study sample

	White British	Indian	Pakistani	Bangladeshi	Caribbean	African
Frequency, *n*	1 61 720	45 070	28 780	83 030	31 667	60 274
Age, years, mean (s.d.)	39.2 (12.2)	36.2 (11.5)	34.9 (11.6)	33.9 (11.3)	41.4 (12.6)	38.8 (12.0)
Gender, male (%)	50.4	56.7	61.2	55.1	42.8	47.7
English main language, %	98.7	54.1	52.2	46.4	99.0	85.4
IMD score, median (interquartile range)	36.8 (15.5)	38.8 (9.5)	40.4 (9.1)	43.6 (13.1)	39.1 (12.9)	41.8 (13.7)
Ethnic density %, median (interquartile range)	36.0 (19.5)	17.4 (23.5)	13.0 (12.8)	29.0 (30.6)	8.8 (6.0)	14.1 (10.0)
Antidepressants prescribed – past year, % (95% CI)^a^	8.1 (8.0–8.3)	2.1 (2.0–2.2)	3.2 (3.0–3.4)	4.4 (4.3–4.6)	3.7 (3.5–3.9)	2.1 (2.0–2.2)
Depression newly diagnosed – past year, % (95% CI)^a^	3.5 (3.4–3.6)	0.9 (0.8–1.0)	1.2 (1.1–1.3)	1.5 (1.4–1.6)	2.2 (2.0–2.3)	1.0 (0.9–1.1)

CI, Confidence interval.

a Adjusted for age and gender only.

**Fig. 1.**
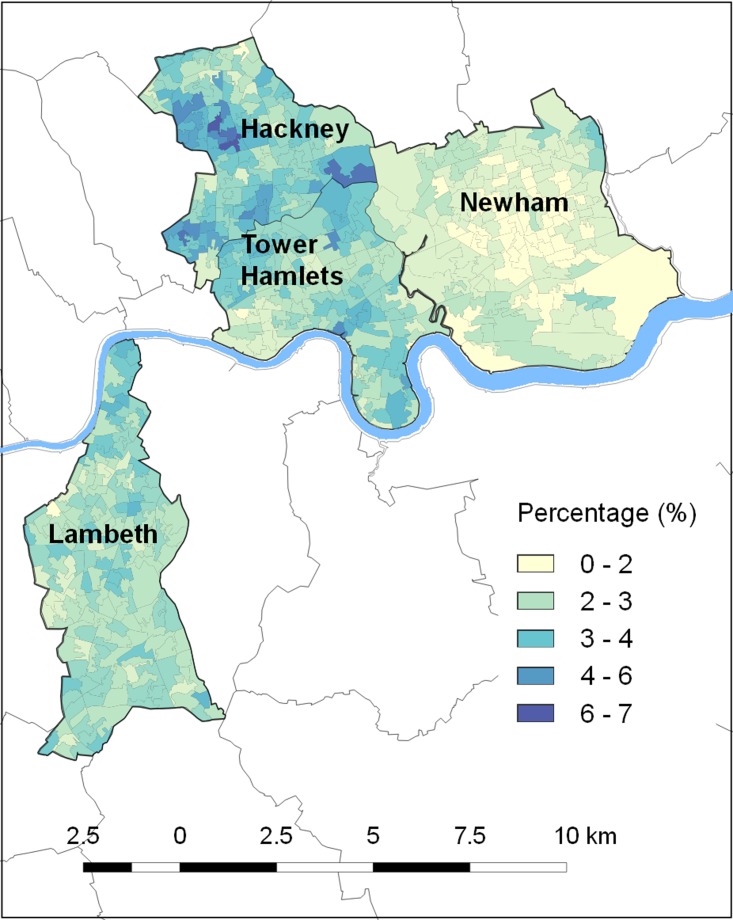
New depression diagnosis in Lambeth and East London – percentage of primary care patients with Quality and Outcomes Framework (QOF) depression code in previous year by neighbourhood (lower super output area).

**Fig. 2.**
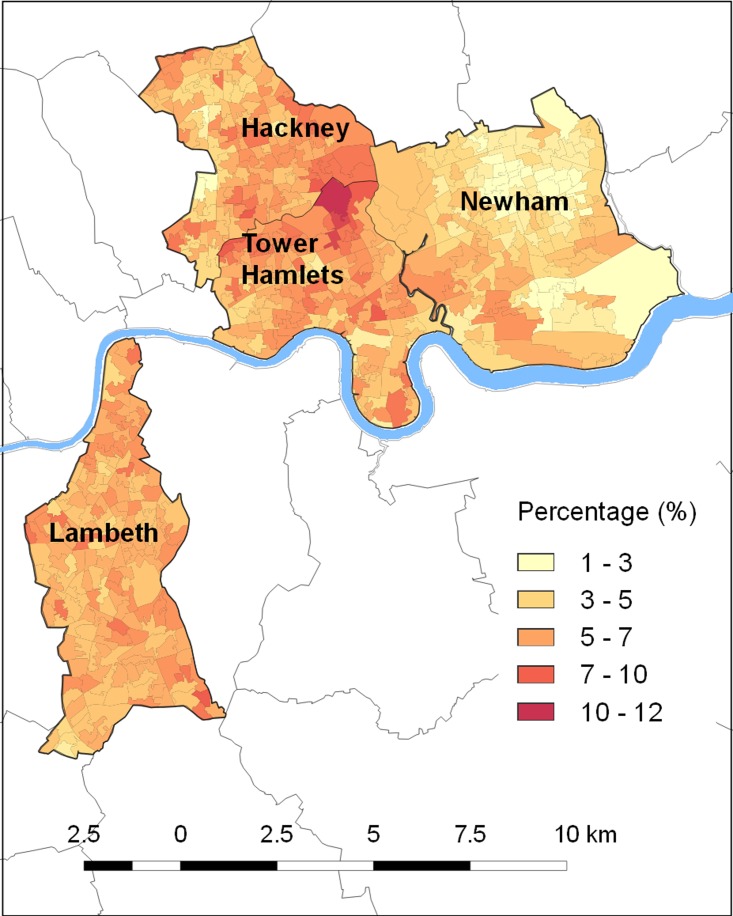
Antidepressant use in Lambeth and East London – percentage of primary care patients prescribed antidepressants by neighbourhood (lower super output area).

Both depression diagnosis and antidepressant use also showed clear spatial patterning, which appears to relate to area ethnic profiles (Figs 1 and 2). For example, both figures show some areas, such as Newham, where these outcomes are rare whereas for other areas, such as parts of Hackney and Tower Hamlets, these are much more common. It is notable that Newham has the lowest proportion of white British residents in our sample, at just under 17% (Census, 2011). Conversely the areas of Hackney and Tower Hamlets with the highest depression diagnosis and antidepressant use have a particularly high proportion of white British residents

Looking at area differences in more detail we found a noticeable decrease in recent depression diagnosis (Table 2), for some ethnic groups, in areas with greater own-ethnic density. After adjusting for area deprivation, age, gender and GP practice, we found that for the Pakistani group the odds of recent depression diagnosis decreased by a factor of 0.81 [95% confidence interval (CI) 0.70–0.93] for each 10% increase in the proportion of Pakistani people in the area. Similar results were found for the Indian [odds ratio (OR) 0.88, CI 0.81–0.95) and African (OR 0.88, CI 0.78–0.99) groups and a small effect for Bangladeshis (OR 0.94, CI 0.90–0.99). For black Caribbeans, however, neighbourhood ethnic density had the opposite effect and was associated with an increase in new depression diagnosis (OR 1.26, OR 1.09–1.46).

**Table 2.** The relation between area ethnic density and depression diagnosis/antidepressant use by ethnic group in Lambeth and East London

	Effect of 10% increase in area ethnic density on …			
	new depression diagnosis (in the past year)		antidepressant use (in the past year)	
Ethnic group	OR (95% CI)^a^	*p* value	OR (95% CI)^a^	*p* value
Indian	0.88 (0.81–0.95)	<0.01	0.85 (0.81–0.90)	<0.01
Pakistani	0.81 (0.70–0.93)	<0.01	0.86 (0.78–0.96)	<0.01
Bangladeshi	0.94 (0.90–0.99)	0.01	0.97 (0.94–1.00)	0.05
Caribbean	1.26 (1.09–1.46)	<0.01	1.10 (0.99–1.23)	0.07
African	0.88 (0.78–0.99)	0.04	0.86 (0.80–0.93)	<0.01

CI, Confidence interval.

a Ratio adjusted for age, gender and area deprivation.

We also found a corresponding decrease in antidepressant use in areas with greater ethnic density, for the same ethnic groups. For Indians, the odds of using antidepressants decreased by a factor of 0.85 (95% CI 0.81–0.90) for each 10% increase in own ethnic density. Similarly antidepressant use decreased proportionally as own-group ethnic density increased for Pakistanis (OR 0.86, 95% CI  0.78–0.96), Africans (OR 0.86, 95% CI  0.80–093) and Bangladeshis (OR 0.97, 95% CI  0.94–1.00). For black Caribbeans neighbourhood ethnic density was, again, associated with the opposite effect: an increase in antidepressant use (OR 1.10, CI 0.99–1.23) in higher ethnic density areas, although this was just outside the criteria for statistical significance (*p* = 0.07).

When we adjusted for English as main language this failed to make any appreciable difference to the effect of area ethnic density and the results remained unchanged whichever way missing data was imputed (see  Supplementary Appendix Tables S1*a*–S1*c*).

## Discussion

### Summary

We found up to a 75% reduction in new depression diagnosis and antidepressant use for some ethnic groups compared to the white British population. This was, in part, related to ethnic density for Indian, Pakistani, Bangladeshi and African groups; with an increase of 10% in area ethnic density corresponding with a decrease in the odds of depression diagnosis by up to 20%. Similarly, for each of these ethnic groups, antidepressant use decreased as ethnic density increased. A more complex picture emerged for black Caribbeans where the opposite effect was observed. Our measure of acculturation made no difference to these observed effects.

### Ethnic differences in common mental disorders

Our results are in line with previous studies showing lower rates of antidepressant use among BME groups. For example, Cooper *et al.* (2013) used national psychiatric morbidity survey data to investigate antidepressant use and found lower overall rates, with 1.8% of black people taking antidepressants compared to 3.5% of white people. Studies of depression diagnosis have shown more mixed results, with some concluding that rates among BME groups are typically lower (Lloyd, 1993) while a more recent international review gives slightly higher rates among ethnic minorities although studies are shown to differ widely (Tarricone *et al.* 2012). This is in contrast to studies looking at more severe mental health problems, where rates have been shown to be consistently higher (Veling, 2013).

### Ethnic density effects

Our findings, showing an ethnic density effect for antidepressant use, mirror those of studies conducted at the GP practice level. For example, Hull *et al*. (2001) found the percentage of Asian names on the practice list explained 30% of the variation in practice prescribing in their East London study. Similarly Walters *et al*. (2008) analysing national GP practice data, found the proportion of black people to be the strongest predictor of prescribed antidepressant volume. By looking at the interaction between individual ethnicity and area ethnic composition we have shown that this effect is retained at the individual level.

Comparing our results with studies looking directly at underlying depression and anxiety there are some strong similarities. For example, Das-Munshi *et al.* (2010) found similar protective ethnic density effects for the equivalent ethnic groups in their analysis of EMPIRIC data, a large (*N* = 3446) national community survey investigating BME mental health. For each 10% increase in own-group ethnic density they found a decrease in rates of common mental disorder for Bangladeshi (OR 0.77, 95% CI 0.66–0.92), Indian (OR 0.77, 95% CI 0.73–1.07) and Pakistani (OR 0.92, 95% CI 0.83–1.02) respondents, after adjusting for area deprivation. As with our study, they failed to find any protective ethnic density effect for the black Caribbean group (*p* = 0.65). Pickett *et al.* (2009) looked at maternal mental health using the Millennium Cohort Study (*N* = 2318 BME respondents) and found protective ethnic density effects for Indian and Pakistani mothers but failed to find a statistically significant effect for Bangladeshi, Caribbean or African mothers. Our results broadly fit the ethnic pattern they observed, with the exception of African mothers which may be because their sample for this group was so much smaller (*n* = 367). Halpern & Nazroo (2000) looked at the same question using a similar national community survey dataset (*N* = 8063) and found Indians showed the strongest correlation between neurotic symptoms and ethnic density (partial *r* = −1.59), after adjusting for age, sex and `hardship’. Contrary to our findings, this was partly attenuated by language which reduced the correlation with ethnic density slightly (*r* = −1.45). As with our study they found a much smaller effect for the Bangladeshi and black Caribbean samples. Conversely, other UK studies have failed to detect any ethnic density effect (Shields & Wailoo, 2002; Propper *et al.* 2005) although this, again, may reflect relatively small BME sample sizes.

Shaw *et al.*'s (2012) comprehensive meta-analysis shows consistent protective ethnic density effects for south Asian groups in the UK but, of the four UK studies of black African and Caribbean populations, only one showed a positive effect. This, they argue, may be because the black population in the UK tends not to be so highly concentrated. This appears to be borne out by the results of our study, where black Caribbeans live in areas with the lowest median ethnic density and this is the one group that failed to show a protective ethnic density effect. That our results showed an *adverse* ethnic density effect may reflect greater deprivation in high black Caribbean density areas that our deprivation measure (IMD scores) has failed to capture. Further research is needed to determine whether this is actually the case. Shaw and colleagues also looked at US studies, where similar protective ethnic density effects are shown for African American, Hispanic and East Asian groups although, as in the UK, these were not necessarily consistent (Shaw *et al.* 2012). Also some US studies report the opposite adverse ethnic density effect for African Americans although it is important to bear in mind that area ethnic composition is very different in the United States making it difficult to directly compare results with the UK (Mair *et al.* 2008; Becares *et al.* 2014).

### Strengths and limitations

This is the largest study to date to examine the relation between neighbourhood factors and depression for different ethnic groups. We were able to show how routinely collected health records could be used to investigate neighbourhood effects on health for a primary-care population, using advanced statistical methods that allowed us to adjust for the effect of GP practice attended. Furthermore, the outcomes we looked at, antidepressant prescribing and new depression diagnosis, are comprehensively coded.

However, it is important to bear in mind that this is administrative data and therefore subject to bias resulting from the administrative processes that generated the data in the first place. While primary-care records are good at showing prescribing patterns, GPs tend to under-record depression, often recording symptoms only (Rait *et al.* 2009; Kendrick *et al.* 2015). This could introduce bias where, for example, an inner-city practice with a high workload might be more inclined to under-record depression to avoid further administrative burden. We set out to address this problem in two ways. First, in the study design, we included an additional proxy measure of depression that is not subject to the same bias as coded diagnosis. We used antidepressants prescribed, as prescribing data is automatically entered into GP practice systems. The fact that our results using both measures are essentially the same suggests that, for the questions we are asking, bias due to inconsistent diagnostic coding is not an issue. Second, in the analysis, we also adjusted for practice level factors that might influence, and therefore possibly bias, both diagnostic coding and antidepressant prescribing. In the statistical model we included GP practice as a separate level cross-classified with the neighbourhood. This allowed us to account for practice level effects, such as any tendency to under-code depression, so that our final results show the effect of living in a particular neighbourhood independent of practice effects.

It is also possible that antidepressants may be prescribed for physical problems, such as for acute pain relief, rather than specifically for mental health problems; and that this may be more common among some ethnic groups. We were though, able to exclude those drugs most commonly used for other indications. Therefore, while the resulting bias cannot be completely ruled out its impact overall is likely to be small. Looking at our results overall the fact that odds ratios for both new diagnosis and antidepressant use are so similar suggests that this source of bias does not represent a serious study limitation.

While we were able to access a very large and comprehensive dataset of health records we acknowledge that one limitation of our sample was that patients aged ⩾65 years were excluded as this was the only data that we had available at the time of the study. However, for most of the ethnic groups we looked at this represents a very small minority (around 6% of the total) and is therefore unlikely to have affected our overall results. The one exception was the black Caribbean population where around 20% were aged ⩾65 years. To address this we therefore conducted a sensitivity analysis using data collected for Lambeth patients aged ⩾65 years that we were able to access retrospectively. We re-analysed the data for the black Caribbean population but this time also including older Lambeth patients and found that that this made no appreciable difference to the results (sensitivity analysis results available on request).

Our findings are, of course, specific to the areas covered and therefore caution is needed when generalizing further afield. This has to be set alongside the advantages of concentrating on one specific area: that we were able to look at almost the entire population, we could account for relevant contextual factors including GP practice, and we could examine effects at a more detailed neighbourhood level than has been possible before. This is particularly important in an urban area, such as London, where localities with very different socioeconomic and ethnic profiles are often close by. This explains why when we re-ran the analysis at the broader MSOA our results were less clear, which mirrors previous work looking at ethnic density and severe mental illness where effects were also much clear at LSOA level (Schofield *et al*. 2011).

However, while GP record data has obvious benefits in terms of scale and accuracy of recording it is still important to bear in mind that this can only tell us about service use rather than directly represent underlying disorder. We were, though, able to account for the role of the GP practice in determining service use by using a statistical modelling strategy to adjust for individual GP practice effects. This leaves the question of patients’ own health behaviour. It is likely that our results reflect both underlying disorder and patient's willingness to go to their GP about mental health problems in the first place. Some of the ethnic differences we have shown may therefore reflect the extent to which different groups somatize underlying mental health problems (Cornwell & Hull, 1998). This in turn will reflect well documented cultural differences in attitudes to mental distress and the extent to which this is stigmatised among different ethnic groups (Littlewood & Lipsedge, 1997; Marwaha & Livingston, 2002; Commander *et al*. 2004; Mallinson & Popay, 2007; Chaudhry *et al*. 2008). We did go some way to investigating this by looking at acculturation, as one possible explanatory factor, although the proxy measure we used did not show any difference. We accept that English recorded as the main language is a crude proxy and it is possible that a more nuanced measure would be more revealing. Given the above considerations, our results are likely to reflect both underlying disorder and patterns of health behaviour in combination. To look at underlying disorder alone requires analysis of community survey data with enough respondents sampled from each ethnic group in question, which is very difficult to achieve to the required scale. Our intention is that the present study, with its advantages of scale and whole population coverage, is viewed alongside community survey research conducted on a smaller scale. Therefore we recommend further research on this topic using community survey data. While the present study has examined ethnicity and ethnic density effects independent of practice level factors we also recommend further research looking at how practice and GP characteristics are related to the ethnic differences reported here.

## Conclusions and implications

We found marked ethnic differences in antidepressant use and depression diagnosis and these in turn were related to neighbourhood ethnic composition. A measure of acculturation did not help explain these differences. While we would be cautious about drawing firm conclusions on the basis of one study this does have potential implications for clinical services. First, it is clear that ethnicity is an important factor in mental health service use, as is proximity to one's own ethnic group, and this should be taken into account when planning service provision. Second, we may need to consider better case-finding and treatment for ethnic minorities in areas where they constitute a minority. Third, if proximity to one's own ethnic group is protective then this could be incorporated in mental health service design. For example, support groups for specific ethnic groups could be set up to harness this protective effect.

## Supplementary material

For supplementary material accompanying this paper visit http://dx.doi.org/10.1017/S0033291715002913. click here to view supplementary material
