# Supplementary material for: Does depression diagnosis and antidepressant prescribing vary by location? Analysis of ethnic density associations using a large primary-care dataset
Source: Psychol Med. 2016 Feb 16;46(6):1321–9. doi: 10.1017/S0033291715002913 (PMC4828938; doi:10.1017/S0033291715002913)
Supplement: Supplementary file 1 [file S0033291715002913sup001.docx]

Appendix Table S1a. The relation between area ethnic density and depression diagnosis / antidepressant use by ethnic group in Lambeth and East London – adjusted for English language proficiency (whether English is main spoken language). Model a) Complete cases only

|  | | Effect of 10% increase in area ethnic density on: | | | | | |
| --- | --- | --- | --- | --- | --- | --- | --- |
|  | | New depression diagnosis (in the past year) | |  | Antidepressant use (in the past year) |  |  |
| Ethnic group | | OR (95% confidence interval)^a^ | P value |  | OR (95% confidence interval)^a^ | P value |  |
| Indian | | 0.90 (0.82 to 0.99) | 0.03 |  | 0.87 (0.82 to 0.94) | <0.01 |  |
| Pakistani | | 0.81 (0.67 to 0.99) | 0.04 |  | 0.85 (0.77 to 0.95) | <0.01 |  |
| Bangladeshi | | 0.95 (0.90 to 0.99) | 0.03 |  | 0.97 (0.94 to 1.01) | 0.10 |  |
| Caribbean | | 1.21 (1.04 to 1.42) | 0.01 |  | 1.04 (0.91 to 1.18) | 0.59 |  |
| African | | 0.87 (0.76 to 0.98) | 0.02 |  | 0.83 (0.76 to 0.91) | <0.01 |  |

^a^ratio adjusted for age, gender and area deprivation

Appendix Table S1b. The relation between area ethnic density and depression diagnosis / antidepressant use by ethnic group in Lambeth and East London – adjusted for English language proficiency (whether English is main spoken language). Model b) Missing language – entered as English *not* main language

|  | | Effect of 10% increase in area ethnic density on: | | | | | |
| --- | --- | --- | --- | --- | --- | --- | --- |
|  | | New depression diagnosis (in the past year) | |  | Antidepressant use (in the past year) |  |  |
| Ethnic group | | OR (95% confidence interval)^a^ | P value |  | OR (95% confidence interval)^a^ | P value |  |
| Indian | | 0.89 (0.82 to 0.96) | <0.01 |  | 0.85 (0.80 to 0.91) | <0.01 |  |
| Pakistani | | 0.84 (0.73 to 0.97) | 0.02 |  | 0.88 (0.80 to 0.97) | 0.01 |  |
| Bangladeshi | | 0.95 (0.90 to 1.00) | 0.03 |  | 0.98 (0.95 to 1.01) | 0.15 |  |
| Caribbean | | 1.24 (1.06 to 1.45) | 0.01 |  | 1.08 (0.95 to 1.24) | 0.24 |  |
| African | | 0.87 (0.78 to 0.97) | 0.01 |  | 0.84 (0.78 to 0.92) | <0.01 |  |

^a^ratio adjusted for age, gender and area deprivation

Appendix Table S1c. The relation between area ethnic density and depression diagnosis / antidepressant use by ethnic group in Lambeth and East London – adjusted for English language proficiency (whether English is main spoken language). Model c) Missing language – entered as English *is* main language

|  | | Effect of 10% increase in area ethnic density on: | | | | | |
| --- | --- | --- | --- | --- | --- | --- | --- |
|  | | New depression diagnosis (in the past year) | |  | Antidepressant use (in the past year) |  |  |
| Ethnic group | | OR (95% confidence interval)^a^ | P value |  | OR (95% confidence interval)^a^ | P value |  |
| Indian | | 0.89 (0.82 to 0.97) | 0.01 |  | 0.86 (0.81 to 0.91) | <0.01 |  |
| Pakistani | | 0.82 (0.73 to 0.92) | <0.01 |  | 0.87 (0.79 to 0.96) | 0.01 |  |
| Bangladeshi | | 0.95 (0.90 to 0.99) | 0.03 |  | 0.98 (0.95 to 1.01) | 0.11 |  |
| Caribbean | | 1.26 (1.02 to 1.55) | 0.03 |  | 1.10 (0.99 to 1.23) | 0.07 |  |
| African | | 0.88 (0.78 to 0.99) | 0.03 |  | 0.86 (0.80 to 0.92) | <0.01 |  |

^a^ratio adjusted for age, gender and area deprivation

Appendix S2 - list of antidepressants excluded from the study:

Amitriptyline

Anafranil

Clomipramine

Cymbalta

Duloxetine

Fluanxol

Flupentixol

Nortriptyline

Tryptizol

Yentreve
